# Supplementary material for: Exhausted T cell signature predicts immunotherapy response in ER-positive breast cancer
Source: Nat Commun. 2020 Jul 17;11:3584. doi: 10.1038/s41467-020-17414-y (PMC7367885; doi:10.1038/s41467-020-17414-y)
Supplement: Supplementary file 1 — Supplementary Information [file 41467_2020_17414_MOESM1_ESM.pdf]

Supplementary Information for

Exhausted T cell signature predicts immunotherapy response in ER-positive breast cancer.

Terranova-Barberio et al.

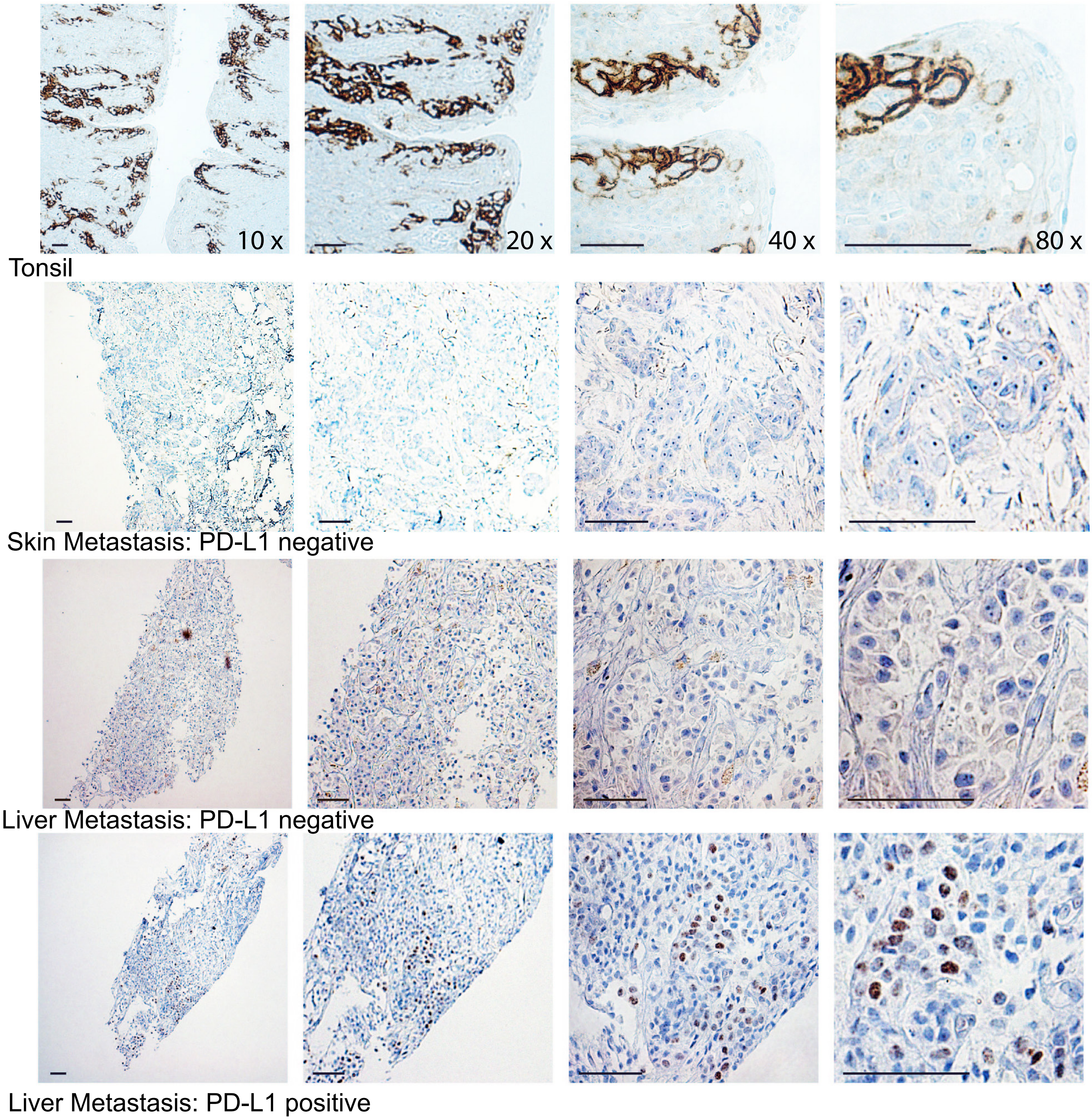

Supplementary Figure 1: Immunohistochemistry analysis of PD-L1 expression on Paraffin-embedded tissues. Tumors were freshly isolated from patients at each time point, when accessible. Tissue sections originated from tonsil (as positive control), skin and liver metastasis were stained and scored for PD-L1 expression (clone SP263). Representative images of each tissue are shown, captured with a 10x, 20x, 40x or 80x objective on a light microscope. Scale bar = 50µm. IHC staining was performed on one section/sample using an automated IHC stainer as part of standard clinical lab procedure.

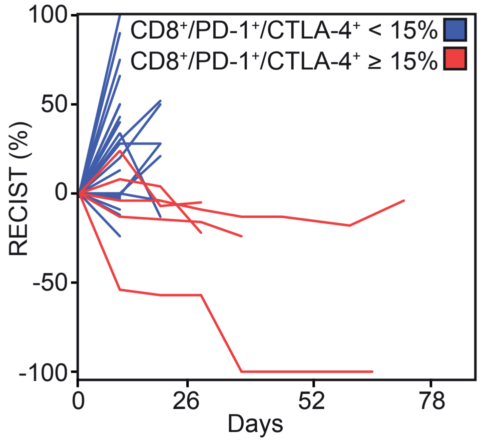

Supplementary Figure 2: Spider plot comparing the survival of patients. Patients with PD-1+CTLA-4+ CD8+ T cells expression in tumor or blood > 15% are shown in red, while those with % <15 are shown in blue. Median survival 8.6 vs 2.8 months, HR 0.32 (log rank), p= 0.0009.

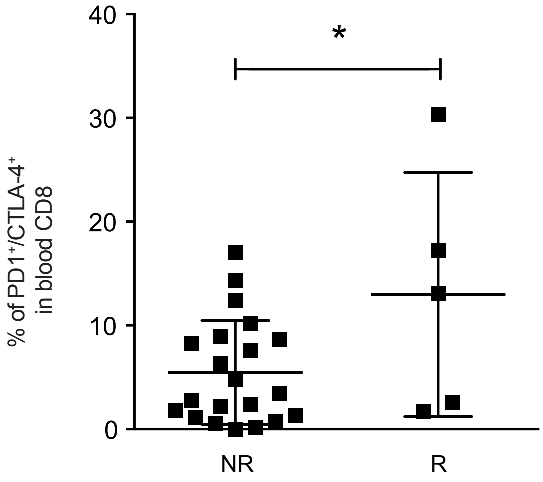

Supplementary Figure 3: Flow cytometry quantification of PD-1+CTLA-4+ CD8+ T-cells in non-responders (NR) versus responders (R) in blood. Data are presented as the mean ± SD (n=26). Statistical significance is indicated by p-values as * P ≤ 0.05; ** P ≤ 0.01; **** P ≤ 0.0001; NS = not significant and was determined by unpaired two-tailed T test with p value = 0.032.

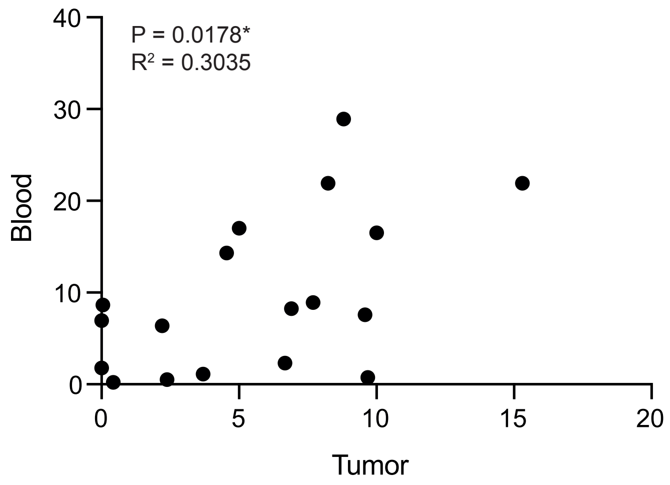

Supplementary Figure 4: Pearson correlation analysis of the relationship between peripheral blood and tumors, performed in non-responders (n=18). Two-sided Pearson correlation test was performed with a p value = 0.0178. Statistical significance is indicated by p-values as * P ≤ 0.05.

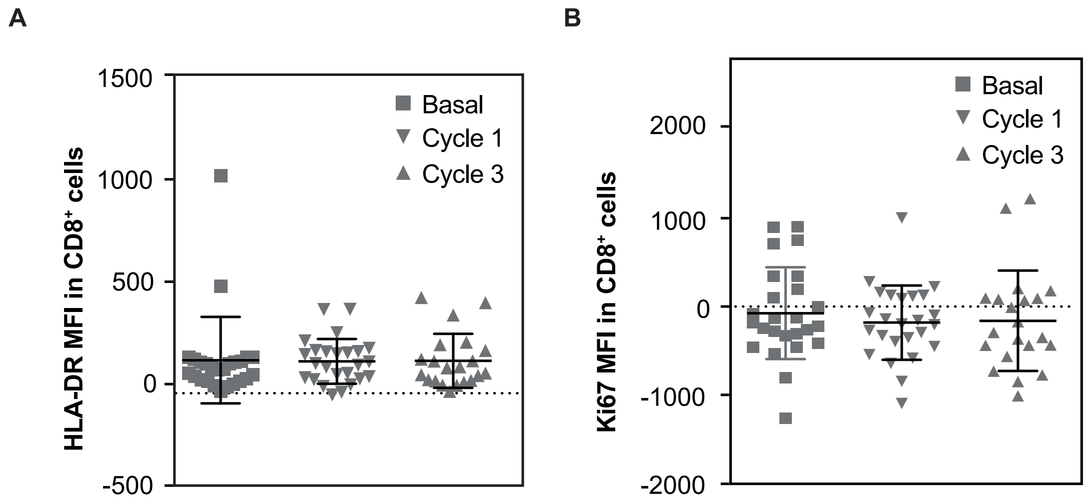

Supplementary Figure 5: Flow cytometric quantification of HLAFlow cytometric quantification of HLADR (A) and Ki67 (B) expression in live CD8+ T-cells from blood at baseline, on day 12 of Cycle 1 and at the end of Cycle 3 (n=25). Data are presented as the mean ± SD. No statistical significance was found in any of the panels and was determined by Kruskal-Wallis test for A and one-way ANOVA for B.

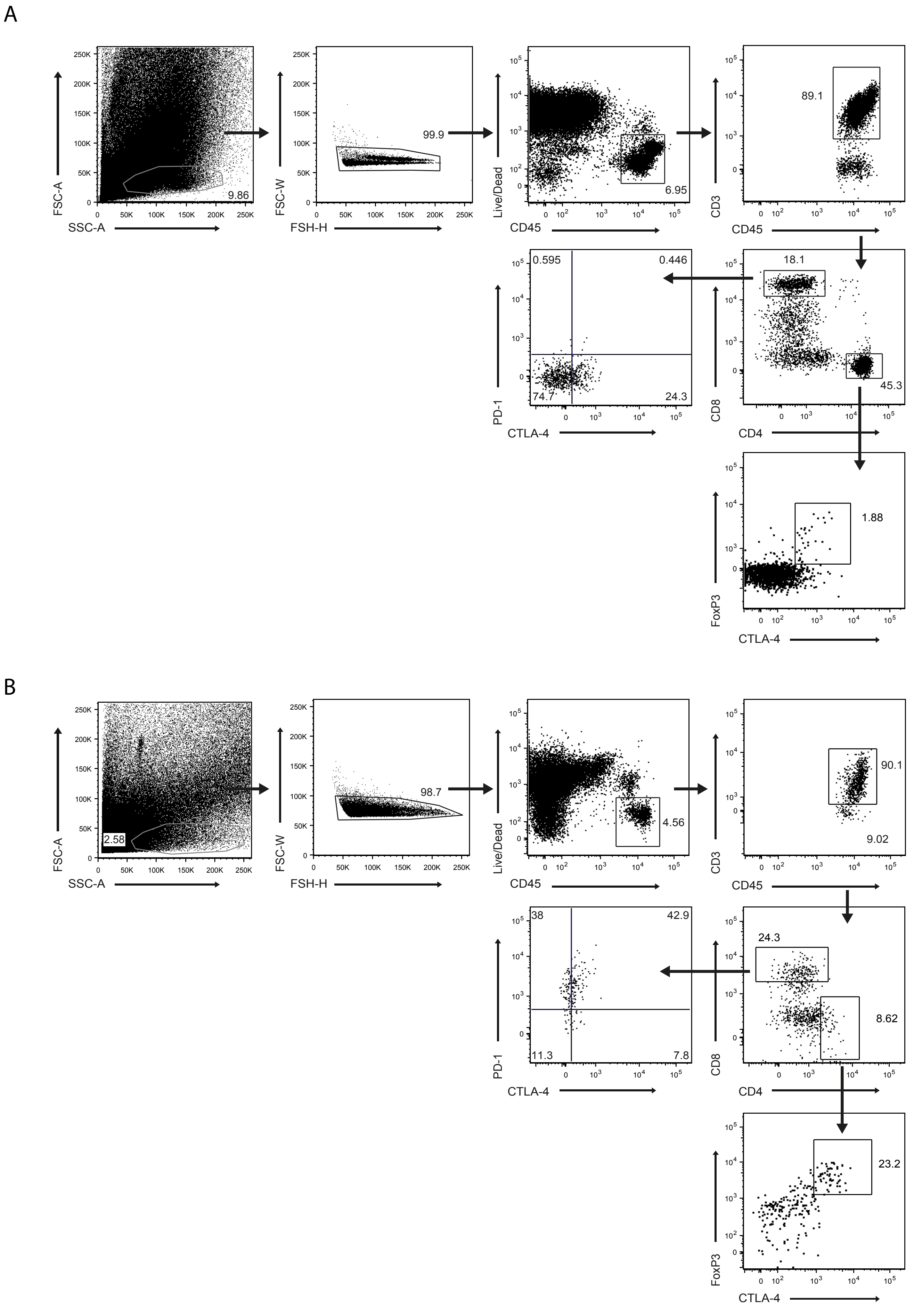

Supplementary Figure 6. Flow cytometry gating strategy. Gating strategy depicting a tumor specimen of a non-responder (A) and a responder (B). For isolation of CD45+ cells (live CD45+), CD3+ cells (live CD45+CD3+), CD4+ Treg cells (live CD45+CD3+CD4+CD8-Foxp3+CTLA-4+), exhausted cytotoxic CD8+ T cells (live CD45+CD3+CD4-CD8+PD-1+CTLA-4+). This gating strategy was used to identify populations described in Figure 2C-D, Figure 3, Figure 4A-C, Figure 5 and Supplementary Figure 3 and Supplementary Figure 5.

Supplementary Table 1.

Immunophenotyping panel		
Biolegend	Cat number	Dilution
anti-CD3	#317324	1:30
anti-PD-1	#329908	1:30
anti-CD45	#304049	1:30
anti-CD8	#301039	1:30
eBioscience		
anti-CD4	#46-0047-42	1:30
anti-CTLA-4	#12-1529-42	1:30
anti-HLA-DR	#47-9956-41	1:30
anti-Foxp3	#48-4777-42	1:30
BD Biosciences		
anti-Ki67	#561277	1:30
Tonbo Bioscence		
Ghost Violet 510 viability dye	#13-0870	1:500

Supplementary Table 1. The immunophenotyping analysis was performed using these flow cytometry
