# Supplementary material for: Exhausted T cell signature predicts immunotherapy response in ER-positive breast cancer
Source: Nat Commun. 2020 Jul 17;11:3584. doi: 10.1038/s41467-020-17414-y (PMC7367885; doi:10.1038/s41467-020-17414-y)
Supplement: Supplementary file 3 — Reporting Summary [file 41467_2020_17414_MOESM3_ESM.pdf]

nature research

Corresponding author(s): Pamela N Munster

Last updated by author(s): Jun 16, 2020

Reporting Summary

Nature Research wishes to improve the reproducibility of the work that we publish. This form provides structure for consistency and transparency in reporting. For further information on Nature Research policies, see Authors & Referees and the Editorial Policy Checklist .

Statistics

For all statistical analyses, confirm that the following items are present in the figure legend, table legend, main text, or Methods section.

n/a	Confirmed	
☐	☒	The exact sample size (n) for each experimental group/condition, given as a discrete number and unit of measurement
☐	☒	A statement on whether measurements were taken from distinct samples or whether the same sample was measured repeatedly
☐	☒	The statistical test(s) used AND whether they are one- or two-sided<br>*Only common tests should be described solely by name; describe more complex techniques in the Methods section.*
☐	☒	A description of all covariates tested
☐	☒	A description of any assumptions or corrections, such as tests of normality and adjustment for multiple comparisons
☐	☒	A full description of the statistical parameters including central tendency (e.g. means) or other basic estimates (e.g. regression coefficient) AND variation (e.g. standard deviation) or associated estimates of uncertainty (e.g. confidence intervals)
☐	☒	For null hypothesis testing, the test statistic (e.g. F, t, r) with confidence intervals, effect sizes, degrees of freedom and P value noted<br>*Give P values as exact values whenever suitable.*
☒	☐	For Bayesian analysis, information on the choice of priors and Markov chain Monte Carlo settings
☒	☐	For hierarchical and complex designs, identification of the appropriate level for tests and full reporting of outcomes
☒	☐	Estimates of effect sizes (e.g. Cohen's d, Pearson's r), indicating how they were calculated

Our web collection on statistics for biologists contains articles on many of the points above.

Software and code

Policy information about availability of computer code

Data collection	Data were collected using BD FACSDiva Software version 8.0 and BD FACSuite v1.0.6.
Data analysis	Data were analyzed using BD FlowJo version 9.9.5. Statistic was performed using GraphPad Software Prism version 8.

For manuscripts utilizing custom algorithms or software that are central to the research but not yet described in published literature, software must be made available to editors/reviewers. We strongly encourage code deposition in a community repository (e.g. GitHub). See the Nature Research guidelines for submitting code & software for further information.

Data

Policy information about availability of data

All manuscripts must include a data availability statement. This statement should provide the following information, where applicable:

- Accession codes, unique identifiers, or web links for publicly available datasets
- A list of figures that have associated raw data
- A description of any restrictions on data availability

All relevant data that support the findings of this study are available from the corresponding author upon reasonable request.

Field-specific reporting

Please select the one below that is the best fit for your research. If you are not sure, read the appropriate sections before making your selection.

☒ Life sciences ☐ Behavioural & social sciences ☐ Ecological, evolutionary & environmental sciences

For a reference copy of the document with all sections, see nature.com/documents/nr-reporting-summary-flat.pdf

Life sciences study design

All studies must disclose on these points even when the disclosure is negative.

Sample size	The trial was initially designed to enroll 87 patients. Using an Optimal Simon's two-stage design, the null hypothesis (H_0) that the true response rate is 10% was to be tested against a one-sided alternative. The trial protocol stated that In the first stage, 10 patients would be accrued and a decision would be made to continue or stop accrual based on the observed objective response rate (ORR). If there were 0 or 1 objective responses at 24 weeks in these 10 patients, accrual to this arm of the study would be stopped. Otherwise, 19 additional patients will be accrued for a total of n=29 with an estimated ORR ≥ 30%. This study was designed to have an alpha of 0.0471, and a power of 81%. As the trial enrolled 34 patients it became clear that unselected patients would not benefit, and a decision was made by the study team and accepted by the institutional review committees that it would not be in the best interest of the patients to continue this study for lack of efficacy. The study was halted early after enrolling 34 of the 87 patients originally planned, due to its limited efficacy in an unselected patient population.
Data exclusions	Of the 34 patients enrolled, 7 were considered not evaluable based on the trial protocol requirements because did not receive permbrolizumab. Six of the 34 patients never received pembrolizumab due to progression prior to scheduled infusion. One patient was taken off study at week 3, without evidence of progression, due to immune-related grade 3 hepatitis. These 7 patients were excluded for the evaluation of clinical responses and in the correlatives studies.
Replication	Immunophenotyping and IHC experiments were performed as a single experiment on freshly obtained PBMCs and tissues from patients at each time point once consent was obtained. IHC was part of standard clinical lab procedure. Observed marker expression patterns in flow cytometry were consistent with previous publications results. For histological staining used in this study, we find that in all cases the expression patterns were consistent with tumor type-specific expectations from previous studies of protein antibody stains.
Randomization	Patients were randomized to either arm using a computerized randomization program by the investigators.
Blinding	This is an open-label randomized trial; therefore, the Sponsor, investigator and subject knew the treatment administered. Two arms were tested, with each independent assessment. Flow cytometry analysis was performed using same gating strategy applied equally to all samples.

Reporting for specific materials, systems and methods

We require information from authors about some types of materials, experimental systems and methods used in many studies. Here, indicate whether each material, system or method listed is relevant to your study. If you are not sure if a list item applies to your research, read the appropriate section before selecting a response.

Materials & experimental systems

n/a	Involved in the study	
☐	☒	Antibodies
☒	☐	Eukaryotic cell lines
☒	☐	Palaeontology
☒	☐	Animals and other organisms
☐	☒	Human research participants
☐	☒	Clinical data

Methods

n/a	Involved in the study	
☒	☐	ChIP-seq
☐	☒	Flow cytometry
☒	☐	MRI-based neuroimaging

Antibodies

Antibodies used	Antibodies used are listed in Supplementary Table 1
Validation	Anti CD3, anti-CD4, anti-CD8, anti-CTLA-4, anti-HLADR, anti-FoxP3, anti-CD45, anti-PD-1, anti-Ki67 antibodies were validated for human use by each vendor and previously used in published studies, including Daud A et al, J Clin Inv 2016, doi: 10.1172/JCI87324

Human research participants

Policy information about studies involving human research participants

Population characteristics	The trial enrolled 18 years or older pre and postmenopausal women or men with stage IV ER+ breast cancer histological or cytological confirmation. Progressed after at least one line of hormonal therapy, any number of prior chemotherapy in the metastatic setting, any number of prior hormonal therapies, HER2 positive or negative. Other inclusion and exclusion criteria are available on the trial protocol.
Recruitment	Patients were enrolled from the clinicians in the study after providing informed consent. Patients were recruited through the UCSF breast program, and self referred by patients reading about the study on cinicaltrials.gov. All patients were offered other trials or standard of care options. Patients were randomly assigned to either arm and both arms contained all three drugs, so no participants in either group dropped out or crossed over.
Ethics oversight	The protocol, the proposed informed consent form and all forms of participant information related to the study were reviewed and approved by the UCSF CHR (UCSF Institutional Review Board) and by the Helen Diller Family Comprehensive Cancer Center Site Committee and by the Protocol Review Committee (PRC).

Note that full information on the approval of the study protocol must also be provided in the manuscript.

Clinical data

Policy information about clinical studies

All manuscripts should comply with the ICMJEguidelines for publication of clinical research and a completedCONSORT checklist must be included with all submissions.

Clinical trial registration	The trial was registered on ClinicalTrials.gov. The Identifier is: NCT02395627 and is accessible via this link: https://clinicaltrials.gov/ct2/show/NCT02395627?term=NCT02395627&draw=2&rank=1
Study protocol	CC #147523
Data collection	Data were collected from May 2015 and January 2017 at UCSF Medical Center at Mount Zion and at UCSF Helen Diller Family Comprehensive Cancer Center. Data were analyzed between July 2015 and January 2018.
Outcomes	Primary Endpoints<br>a) Overall response rate (CR+PR and SD at 24 weeks)<br>b) Toxicity<br>Secondary Endpoints<br>a) Duration of response rate<br>b) 24 week landmark progression free survival (PFS:24),<br>c) Median PFS and overall survival (OS),<br>d) Tumor responses calculated by Immune Related Response-Criteria (irRC)<br>e) Test the response of PD-L1 expression to epigenetic modulation

Flow Cytometry

Plots

Confirm that:

- [x] The axis labels state the marker and fluorochrome used (e.g. CD4-FITC).
- [x] The axis scales are clearly visible. Include numbers along axes only for bottom left plot of group (a 'group' is an analysis of identical markers).
- [x] All plots are contour plots with outliers or pseudocolor plots.
- [x] A numerical value for number of cells or percentage (with statistics) is provided.

Methodology

Sample preparation	Freshly isolated samples were minced and digested overnight with buffer consisting of collagenase type IV (Worthington Biochemical Corp., #4188;), DNAse (Sigma-Aldrich, #DN25), 1% penicillin-streptavidin (CCF, #INVZR124), 1% 1nM Na-pyruvate (Millipore, #TMS-005-C), 1% HEPES (Gibco, #15630-080), 1x non-essential amino acids (Gibco, #11140-050) and 11nM-mercapto-ethanol (Sigma-Aldrich, #M6250) in RPMI medium (Gibco, # 21870092) and collected as single-cell suspensions. PBMCs were suspended in 10% FBS, 1% HEPES, and 1% penicillin-streptavidin in RPMI medium. Approximately 3x106 cells were characterized by a multiparameter flow cytometry analysis performed on a BD LSRFortessa and analyzed by FlowJo software (BD Biosciences).
Instrument	BD LSRFortessa

Software	Data were acquired with BD Diva software and analyzed with BD FlowJo software
Cell population abundance	N/A No sorts were performed.
Gating strategy	Gating strategy is provided in Supplementary Figure 4. From SSC and FSC, singlets Live CD45+ cells were isolated, then CD3+ and then CD4 and CD8+cells were isolated from the CD3. Foxp3, CTLA-4, PD-1 expression was then measured on isolated populations.

☒ Tick this box to confirm that a figure exemplifying the gating strategy is provided in the Supplementary Information.
